# A strategic analysis of health behaviour change initiatives in Africa

**DOI:** 10.1080/16549716.2023.2202931

**Published:** 2023-05-02

**Authors:** Ebele R.I. Mogo, Shaayini Shanawaz, Oreoluwa Ademola-Popoola, Neelam Iqbal, Osazemen Aghedo, Muili Ademola, Nnenna Onyemaobi, Aderayo Eniayewun, Babatunde Ademusire, Tomiwa Adaramola, Adaobi Ugwu, Adaora Obi, Anthony Lerno, Jaachimma Nwagbara, Aimable Uwimana, Elias Gbadamosi, Ajoke Adebisi, Binta Sako

**Affiliations:** a ERIM Consulting, Lagos, Nigeria; b University of Waterloo, Waterloo, ON, Canada; c College of Medicine, University of Ibadan, Ibadan, Nigeria; d Institute of Infection, Veterinary and Ecological Sciences, University of Liverpool, Liverpool, UK; e Faculty of Movement and Rehabilitative Science, Katholieke Universiteit Leuven, Leuven, Belgium; f Department of Public Health, University of Ibadan Oyo State, Ibadan, Nigeria; g Engage Africa Foundation, Alberta, Canada; h Faculty of Education, Nnamdi Azikiwe University, Awka, Nigeria; i Stobhill Hospital, Greater Glasgow and Clyde, Glasgow, UK; j Engage Africa Foundation, Waterloo, Canada; k Engage Africa Foundation, Kigali, Rwanda; l Colorado State University, Fort Collins, Colorado, USA; m Engage Africa Foundation, Abuja, Nigeria; n Tobacco and Other NCD risk factors Team, Universal health Coverage/Healthier Populations, Inter Country Support Team for West Africa, WHO Regional Office for Africa, Brazzaville, The Republic of the Congo

**Keywords:** Behaviour change, Africa, prevention, systems change, health initiatives

## Abstract

**Background:**

Changed health behaviours can contribute significantly to improved health. Consequently, significant investments have been channelled towards health behaviour change initiatives in Africa. Health behaviour change initiatives that address social, economic and environmental levers for behaviour change can create more sustained impact.

**Objectives:**

Through a scoping study of the literature, we explored the literature on behaviour change initiatives in Africa, to assess their typologies. We explored whether the availability of initiatives reflected country demographic characteristics, namely life expectancy, gross domestic product (GDP), and population sizes. Finally, we assessed topical themes of interventions relative to frequent causes of mortality.

**Methods:**

We used the Behaviour Change Wheel intervention categories to categorise each paper into a typology of initiatives. Using Pearson’s correlation coefficient, we explored whether there was a correlation between the number of initiatives implemented in a country in the specified period, and socio-demographic indicators, namely, GDP per capita, total GDP, population size, and life expectancy.

**Results:**

Almost 64% of African countries were represented in the identified initiatives. One in five initiatives was implemented in South Africa, while there was a dearth of literature from Central Africa and western parts of North Africa. There was a positive correlation between the number of initiatives and GDP per capita. Most initiatives focused on addressing sexually transmitted infections and were short-term trials and/or pilots. Most initiatives were downstream focused e.g. with education and training components, while upstream intervention types such as the use of incentives were under-explored.

**Conclusion:**

We call for more emphasis on initiatives that address contextual facilitators and barriers, integrate considerations for sustainable development, and consider intra-regional deprivation.

## Introduction

Several development goals are contingent on changed behaviours. For example, the health-related Sustainable Development Goals require changes in practices around nutrition, smoking, waste management, physical activity, violence reduction, and healthcare utilisation, to mention but a few [1].

Health behaviour initiatives are defined as those that prevent the development or worsening of illness, prevent mortality and promote health and wellbeing [2]. For example, infectious disease transmission and morbidity can be largely curtailed through sanitation and hygiene initiatives to control their spread [3] as well as the adoption of preventive solutions such as healthy nutrition and adherence to curative therapies e.g. vaccination [4]. Similarly, a whopping 80% of non-communicable diseases (NCDs) can be prevented through the implementation of initiatives addressing underlying behavioural risk factors, particularly physical activity, diet and behaviours contributing to environmental exposures [5].

For sustainable behaviour change, initiatives that intersect infrastructural, environmental and social drivers of behaviours are crucial [6]. At the community level, such initiatives may address cultural practices and social customs. For example, community-level stepwise educational interventions to build trust, address pain points, and openly discuss considerations behind parental vaccine hesitancy have been helpful in changing attitudes and perceptions of vaccine safety [7]. At the neighbourhood level, leverage points for addressing behaviours include the design of built and social infrastructure, for example, addressing opportunities for active travel. At the policy level, governments can introduce instruments to stimulate and encourage behaviour change. For example, policies improving access to healthcare, psychosocial support, transport, and nutrition can play a significant role in people’s adherence to cancer treatment [8]. Promoting the use of clean and renewable energy sources by families and organisations can help prevent respiratory and heart diseases like asthma [9]. Similarly, policy investments in tobacco cultivation as a revenue-generating economic activity can reduce access to land for the cultivation of healthy food [10]. Policies shaping public spaces to better improve the quality of lives of those who use them [11] can overcome entrenched neighbourhood barriers to participation in healthy living [12].

Through a systems lens that incorporates the holistic drivers of behaviours, and a life-course lens, which addresses levers for improved health from childhood to adulthood and ageing, initiatives to promote healthy behaviours may simultaneously address multiple types of diseases. For example, the incidence of diabetes mellitus (DM) has been associated with dietary habits, particularly markers of childhood malnutrition [13]. Meanwhile, DM is a risk factor for hospitalisation and death from infections [14]. Initiatives primarily targeted at reducing the incidence of DM will also reduce the incidence and severity of associated illnesses such as respiratory infections which account for an estimated 10% of deaths in Africa [15,16].

Therefore, health behaviour initiatives should not only aim to inform people about healthy behaviours but also empower people to maintain healthy behaviours and provide an environment that makes practising healthy living sustainable. Through this scoping study, we have taken this lens into consideration and seek to systematically evaluate the landscape of initiatives targeting behaviour change for health in Africa to understand their typology and focus. To achieve this, we have analysed peer-reviewed literature on health behaviour change initiatives implemented in African countries between 2001 and 2021. We categorised these initiatives using a typology of their features. We assessed these features in light of the multi-faceted drivers of health behaviours. We examined the extent to which the landscape reflects an emphasis on long-term against short-term projects. We explored the alignment between prominent behaviours being targeted and country socioeconomic factors. Finally, we explored the implications of our findings for investments in health behaviour change in Africa.

### Research questions

**Our research questions are the following**:

- How is the published literature on health behaviour change initiatives spread across Africa?
- What settings and intervention types are most common in the identified initiatives?
- What is the regional and socioeconomic spread of the identified initiatives?
- Comparatively, how does the literature reflect short-term versus long-term sustainable investments in behaviour change?

## Methodology

### Inclusion and exclusion criteria

We conducted a systematic search for primary quantitative and qualitative peer-reviewed literature on health behaviour change initiatives in Africa. We sought out studies conducted in member states of the African Union and published since 2001. This period was deemed a reasonable estimate for the range of a generation, in line with 15–20-year periods being a typical generational span [17]. Studies that reported initiatives that did not take place in an African country, did not target behaviours, and were implemented before 2001 were excluded. Literature reviews, commentaries, and opinion pieces were also excluded. Initiatives included in this study were required to target changes in health behaviours. These include initiatives that promote and address physical activity, healthy diets, substance use, safe sexual practices, adherence to disease management, healthcare-seeking and waste management, to mention but a few. Initiatives could encourage change, increased adoption, or maintenance of targeted behaviours. They could target any level of the social gradient of health including individual perceptions, attitudes and beliefs; social and interpersonal norms; environmental design and influences; as well as upstream policies, laws, and political will.

### Types of initiatives

We categorised each initiative represented as fixed or continuous. Fixed projects represented short-term projects such as trials and pilots or donor-funded interventions running for a limited time e.g. a 12-month trial. Continuous projects represented initiatives for which the study constituted ongoing learning on a sustained project, such as an impact evaluation of a scaled-up project. We included all outcomes for initiatives that met the inclusion criteria listed above, including quantitative and qualitative outcomes, and infectious and non-infectious diseases.

### Types of participants

We did not have any exclusion criteria for the gender, ethnicity or age of the groups included in this scoping review.

### Literature search

Training for the study was through an online workshop. This provided opportunities to discuss and refine the aims and focus. The study team brought a wealth of policy, practice and community-engaged experience in public health, healthcare and medicine; as well as lived experience in African countries including Nigeria, Kenya, South Africa, and Cameroon to mention but a few. A wide range of experiences in addressing health behaviours, from the individual clinical context to the policy and investment context, was therefore represented. This enriched the search, analysis, and interpretation of findings.

Team members engaged in the brainstorming of key search terms across health behaviour change themes e.g. sexual health; behaviour change constructs e.g. attitudes; grades of change e.g. adoption; and types of health behaviour change initiatives e.g. incentives. These inputs informed the search terms, which were further developed in PubMed using medical subject headings and text words. The search was then adapted to CINAHL and SCOPUS to allow us to capture literature from the social sciences. We did not have language restrictions.

### Study screening and selection

**Figure 1.**
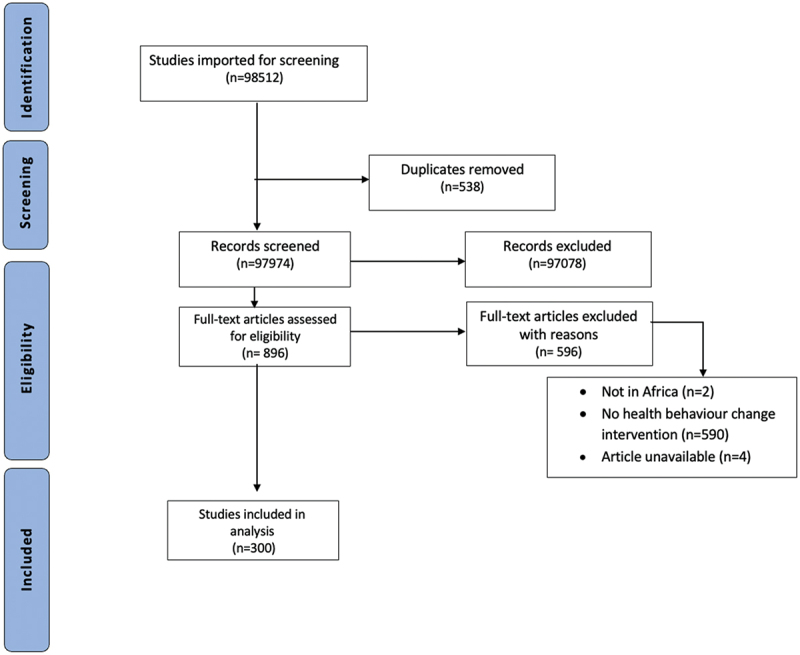
Flowchart for the scoping search.

We exported our search results into Rayyan for further review. Teams double-screened 1% of the studies and subsequently their full texts. Where conflicts arose, inclusion and exclusion criteria, including definitions of initiatives/exposures and outcomes were reviewed, clarified, and refined until conflicts were resolved. Subsequently, 10% of studies were simultaneously reviewed by two reviewers, who clarified any conflicts. Finally, the remainder of the titles and abstracts, and full-text articles, were independently screened. The iterative process (initial screening of 1%, then 10%) allowed for two rounds of iterative learning for the team. We chose this approach to allow for multiple levels of revision and clarification, given that our search was agnostic to outcomes and behaviours and thus broad. Figure 1 below shows the flowchart for the scoping search.

### Data extraction

Articles that met the inclusion/exclusion criteria were extracted. The first author piloted a data extraction template with the second author, via doubly extracting 5% of the articles. Their responses were used to modify the data extraction template where necessary. Researchers were assigned the remainder of the articles to extract, making use of the revised data extraction template. 10% of selected data fields in the final articles were doubly extracted, with concordance checked. The first author also reviewed the extracted data when completed to ensure the completion of the extraction template.

We collected the following information: i) behaviours targeted and typology of behaviour change initiatives according to the BCW [18], ii) whether or not a government policy had supported the specified behaviour change initiative, iii) nature of the initiatives i.e. fixed or continuous projects, iii) settings of the initiatives, iv) country and town of the initiative, and v) populations targeted by initiatives. Team members worked on online versions of documents, enabling collaboration across various locations. Our extraction template is appended to this paper and presents a more extensive description of the information collected in this process.

During the process, the extraction template was updated to capture the typology of behaviour change initiatives based on the Behaviour Change Wheel (BCW) [19]. We used the intervention functions of the BCW to categorise interventions because it allowed us to delineate their characteristics and categorise multi-functional interventions such as those with upstream and downstream components. Additionally, the BCW has been applied to a prolific range of behaviours across infectious and non-communicable diseases [18,20]. We focused on the nine intervention categories of the BCW model, namely: education, persuasion, incentivisation, modelling, coercion, training, restriction, enablement, and environmental restructuring.

The BCW category of education concerns increasing knowledge around behaviour change e.g. if the intervention provided information on healthy eating. Persuasion concerns communicating positive or negative feelings to stimulate action e.g. imagery to motivate an increase in physical activity. Incentivisation involves creating an expectation of a reward e.g. prize draws to induce attempts to stop smoking. Coercion involves creating an expectation of cost or punishment e.g. increasing financial costs to reduce excessive alcohol consumption. Training interventions involved those that imparted skills to participants e.g. advanced driver training to increase safe driving. Restriction involves reducing opportunities for unwanted behaviours and increasing opportunities for acceptable behaviours e.g. prohibiting alcohol sales to people under 18 years. Environmental restructuring involves modifying the physical or social context e.g. using sidewalks to encourage walking. Modelling involves providing an example for people to imitate e.g. using TV shows to promote safe sexual practices like condom use. Enablement involves increasing capability and reducing barriers e.g. support services for smoking cessation.

Using the BCW intervention categories, we categorised each paper into a typology of health behaviour change initiatives. After each researcher coded the BCW intervention categories and assigned articles to initiative typologies, the first author reviewed team members’ input at random, and any potential conflicts of understanding were clarified. Additionally, team members reviewed other team members’ entries at random to see if they agreed with the coding that had been assigned. Recommended changes were proposed between the coding pairs, who had the opportunity to agree with the proposed changes and update their entries or defend their original entries. This input allowed them to learn from one another, revising their entries before the next round so that entries were better aligned. Where there was a dispute that they could not resolve, the first author made the final decision. This process continued until neither of the coding pairs would code the others’ randomly selected entries differently.

## Results

**Figure 2.**
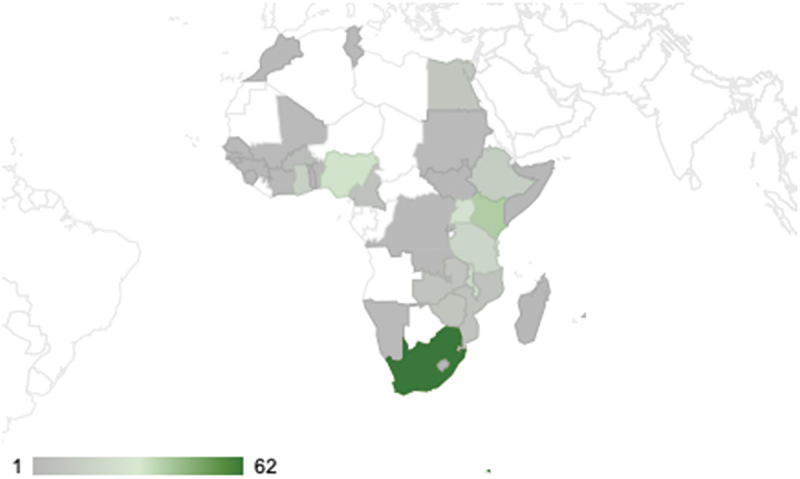
The geographical spread of identified health behaviour change initiatives in Africa.

Figure 2 shows the geographical spread of the identified health behaviour change initiatives, while Table 2 summarises their intervention types. Only 35 African countries, constituting 63.6%, were represented in the peer-reviewed literature. Most of the interventions were implemented in South Africa (19.8%), Kenya (12.5%) and Nigeria (10.5%). Initiatives were most concentrated in Southern and Eastern Africa, and the southern parts of West Africa. Gaps in the literature were most salient in the Central African region as well as the western parts of North Africa.

Using Pearson’s correlation coefficient, we explored whether there was a correlation between the number of health behaviour change initiatives implemented in a country in the specified period, and some socio-demographic indicators, namely, gross domestic product (GDP) per capita, total GDP, population size, and life expectancy (see Table 1).

**Table 2.** Intervention types, percentages and examples.

Intervention type	Percentage	Example
Education: increasing knowledge around behaviour change	61.5%	A peer-led HIV/AIDS and STI health intervention aimed to provide information on safe sexual practices and STIs to prisoners that would soon be released in South Africa [25]. It aimed to reduce risky sexual behaviours, improve their knowledge about sexually transmitted diseases and increase their intention to abstain from risky sexual behaviours.
Training: imparting skills to participants	27.2%	A training program in Kiboga, Uganda, aimed to improve access to information on HIV prevention by training traditional healers to integrate HIV prevention and family planning into their practice [26]. The training supported them to connect family planning, condom use, and HIV prevention and to integrate messages on HIV prevention and family planning into their clinical practices.
Persuasion: communicating positive or negative feelings to stimulate action	26.2%	An intervention used entertainment via a program called ‘Journey to Life in Ethiopia’ to educate the public on HIV [27]. Through an effective entertainment-based education which increased listenership, the intervention aimed to persuade individuals and enable attitude changes, increased knowledge about HIV, and the desire to change behaviours related to the contraction and spread of HIV.
Modelling: providing an example for people to imitate	9.0%	An initiative aimed to improve the effectiveness of an iron-supplementation program to address anaemia during pregnancy. It made use of a community-based strategy that identified and involved positive deviants among pregnant women in rural Senegal [28]. Positive deviants in this case refers to pregnant women who were already adopting beneficial practices thus allowing them to have a higher quality of life and better health. These individuals acted as models to their peers and supported them in practicing affordable and sustainable behaviours and strategies to increase their wellbeing.
Coercion: creating an expectation of cost or punishment	0.0%	None reported
Incentivization: creating an expectation of a reward	4.3%	An intervention in Njombe and Tabora, Tanzania provided either a lottery ticket or a transport voucher as an incentive for encouraging voluntary male medical circumcision [29]. It used a discrete choice experiment in order to evaluate preferences around both possible incentives, as well as the relationship between the choice of incentive and the enrolment of people with more risky sexual behaviours.
Restriction: reducing opportunities for unwanted behaviours and increasing opportunities for acceptable behaviours	0.0%	None reported
Enablement: increasing capability and reducing barriers e.g. support services for smoking cessation.	48.8%	A project provided cognitive behavioural rehabilitation as a form of psychotherapy to sex offenders [30]. The goal of the intervention was to reduce violent sexual behaviours among people who had already committed sexual crimes in South-Eastern Nigerian prisons.
Environmental restructuring: modifying the physical or social context.	7.3%	An action-research project implemented in Western Kenya aimed to establish a school-based horticulture programme to improve the intake of traditional vegetables [31]. At the same time, it aimed to improve pupils’ competence in effecting change and diffusing knowledge in culturally compatible ways.

**Table 1.** Country socioeconomic characteristics vis a vis the proportion of health behaviour change interventions represented by each country.

	PERCENTAGE OF INITIATIVES	GDP PER CAPITA	POPULATION SIZE	LIFE EXPECTANCY	GROSS GDP
South Sudan	0.32%	1,119.70	11,193,725	58	11,997.80
Guinea Bissau	0.32%	813	2,066,233	59	1,638.52
Togo	0.32%	992.3	8,278,724	61	8,413.20
Madagascar	0.32%	514.9	27,691,018	67	14,637.40
Eswatini	0.32%	4,214.90	1,160,164	61	4,941.37
Rwanda	0.32%	833.8	12,952,218	69	11,070.36
Guinea	0.32%	1,174.40	13,132,795	62	15,850.52
Somalia	0.32%	445.8	15,893,222	58	7,292.72
Mauritius	0.32%	8,812.10	1,271,768	74	11,156.66
Lesotho	0.32%	1,166.50	2,142,249	55	2,518.47
Benin Republic	0.64%	1,428.40	12,123,200	62	17,785.64
Morocco	0.64%	3,496.80	36,910,560	77	132,725.26
Sierra Leone	0.64%	515.9	7,976,983	55	4,200.38
Tunisia	0.64%	3,924.30	11,818,619	77	46,840.04
Cote D’Ivoire	0.64%	2,578.80	26,378,274	58	69,764.83
Senegal	0.64%	1,606.50	16,743,927	68	27,625.39
DRC	0.64%	584.1	89,561,403	61	53,958.57
Sudan	0.96%	764.3	43,849,260	66	34,326.06
Namibia	0.96%	4,729.30	2,540,905	64	12,236.25
Senegal	0.96%	1,606.50	16,743,927	68	27,625.39
Mali	0.96%	917.9	20,250,833	60	19,143.74
Burkina Faso	1.28%	918.2	20,903,273	62	19,737.62
Cameroon	2.24%	1,661.70	26,545,863	60	45,238.61
Mozambique	2.56%	500.4	31,255,435	61	16,095.83
Zambia	2.88%	1,120.60	18,383,955	64	21,203.06
Zimbabwe	3.19%	1,737.20	14,862,924	62	26,217.73
Egypt	3.19%	3,876.40	102,334,404	72	404,142.77
Ethiopia	4.47%	944	114,963,588	67	111,271.11
Ghana	5.11%	2,445.30	31,072,940	64	77,594.28
Tanzania	6.07%	1,135.50	59,734,218	66	67,775.10
Malawi	6.39%	642.7	19,129,952	65	12,626.72
Uganda	8.31%	858.1	45,741,007	64	40,434.70
Nigeria	10.54%	2,085.00	206,139,589	55	440,776.97
Kenya	12.46%	2,006.80	53,771,296	67	110,347.08
South Africa	19.81%	6,994.20	59,308,690	64	419,946.43
**Correlation with number of Initiatives**		**0.268169664**	**0.506932201**	**−0.002936374**	**0.679542539**

We wished to explore the following questions: i) whether the availability of health behaviour change initiatives was related to need, and if so, if there would be a correlation between their quantity and the life expectancy of the countries targeted ii) whether economic productivity could potentially be the primary driver of the presence of initiatives in a country instead of the supposed need of its citizens, iii) whether larger population sizes would be related to a higher number of health behaviour change initiatives.

Table 1 above shows our findings on the relationship between these country characteristics and the number of health behaviour change initiatives. Notably, we found a very weak negative correlation between the number of initiatives and life expectancy (−0.0029), a weak positive correlation with population size (0.27), a slightly positive correlation with GDP per capita (0.51), and a strong positive correlation between the number of initiatives and total GDP (0.68).

Healthcare facilities were the modal setting for interventions, followed by community contexts such as markets, neighbourhoods, places of worship, shebeens, and schools. Less represented settings included offices, prisons, and pharmacies. One example of an intervention that was implemented within healthcare settings targeted Nigerian health workers’ attitudes, knowledge, skills and confidence in dealing with patients with the human immunodeficiency virus (HIV) [21]. This initiative used a longitudinal randomised trial, which disseminated information on clinical management, health education, and attitudinal change towards patients with HIV in the intervention group. It aimed to increase the willingness of health workers to treat patients and teach their colleagues about people with HIV, in order to improve their care. Additionally, it worked to increase health workers’ understanding of rights issues in the clinical and psychosocial context which could impact the treatment and prevention of HIV.

An example of an intervention at the community level aimed at improving the knowledge and attitudes that women had about safe abortion in Oromia, Ethiopia [22]. Education on reproductive health and rights was provided within the community setting, building on already ongoing interventions in the community and involving health staff as well as providers in community mobilisation efforts.

We categorised each initiative represented as fixed or continuous. While duration was an important factor to consider, and this was clear in the case of fixed-term pilot tests, a crucial and distinctive consideration was identifying whether the project formed the part of a longer-term or sustainable effort e.g. the scale-up of an intervention or its integration into existing services, or not. We termed ‘fixed projects’ as short-term projects such as trials and pilots or grant-funded interventions running for a limited time, where there was no explicit mention of the project’s continuity. ‘Continuous projects’ on the other hand, represented sustained initiatives for which the study constituted ongoing learning, such as an impact evaluation of a scaled-up project. 74.1% of the studies were fixed projects while 24.6% were continuous.

Fixed-term projects were often time-limited experimental projects. An example of this is a clinical trial that targeted the use of long-term contraceptives among HIV-serodiscordant and concordant couples in Zambia [23]. As part of the intervention, couples were provided with counselling, testing and education through a video format which also modelled future family planning decisions for them. Meanwhile, continuous projects often involved government-run or multi-stakeholder collaborations such as an environmental health initiative run through partnerships between government and non-governmental organisations in four districts in Egypt [24]. This project investigated how people’s behaviours changed within the context of an integrated intervention including safe water supply, sanitation, and education on hygiene. It linked environmental health education with the supply of sanitation services. A continuous learning model was used to evaluate the programme by assessing community behaviours at baseline, and then using household surveys with checklists for key behaviours, namely improved personal hygienic behaviours, and improved handling of drinking water. In comparing baseline, mid-term and final surveys over three years, while the proportions of households that changed behaviours differed in various districts, behavioural improvements were evident in most households in each district.

Where available, we also noted where governmental policies supported initiative provision. Approximately 5% of the interventions involved a government policy. An example of an intervention that involved a policy was the African Youth Alliance program which targeted improvements in the sexual behaviour of young people in Uganda [32]. It was a multifaceted intervention that involved the use of behaviour change communication, the provision of youth-friendly clinical services, institutional capacity building, linkages between policy and advocacy, and the establishment of inter-agency mechanisms to implement youth-focused programs. Through collaborating and networking with government officials and partners, the African Youth Alliance program complemented and scaled up many governmental sexual and reproductive health interventions, hence, widening their coverage and scope.

Approximately 59% of the interventions involved multiple intervention typologies, often combining upstream and downstream interventions. An example is the national scale-up of the community-based distribution of family planning services to improve contraceptive use in Malawi [33]. This project aimed at improving the use of contraception by sexually active young women in the rural areas of Malawi. It was the scale-up of a previously successful pilot. As part of the project, the government targeted the demand for contraceptives and their supply in rural areas by improving the distribution of family planning services. In addition to improving knowledge via already established social structures in the community, this project addressed enablement by removing spatial and financial barriers to accessing services.

Finally, we categorised each paper into an intervention theme to identify the modal health behaviour change themes being targeted. To do so, we identified the target outcome for each initiative, and coded them, aggregating similar codes. This was done iteratively, creating key themes out of the different codes until we arrived at reasonably distinct categories e.g. sexual health, nutrition, and injury prevention. 40.7% of initiatives across the continent were focused on themes of sexual health, particularly the prevention and management of sexually transmitted infections and primarily HIV/AIDS. This was followed by interventions with a focus on improving nutrition (12.9%). In South Africa alone, 56.5% of the initiatives we reviewed also focused on sexual health, particularly the prevention and management of sexually transmitted infections and primarily HIV/AIDS.

## Discussion

Through this scoping study, we sought to systematically evaluate the landscape of initiatives targeting behaviour change for health in Africa. We aimed to understand the nature of this landscape to elucidate the behaviours targeted by such initiatives and whether or not they are aligned with the causes of illness. Additionally, by analysing their typologies, we aimed to assess whether or not these initiatives reflect a socio-ecological understanding of health.

Firstly, the study findings emphasise a positive correlation between each country’s total number of initiatives and total GDP, with South Africa implementing the greatest number of initiatives. Secondly, the findings suggest that most of the initiatives across the continent are primarily focused on sexual health and the management and prevention of sexually transmitted infections, primarily, HIV/AIDS. Thirdly, results suggested that most interventions involved multiple intervention typologies and that education and training were the most common. The findings will be further discussed below.

Our findings showed that interventions were predominantly education and training-focused, which indicates that most documented behaviour change initiatives in Africa do not adequately consider social and contextual barriers which are key drivers of behaviours [34]. This is in line with past research that draws attention to the paucity of contextual considerations in the behaviour change literature in Africa [35]. With the context of health behaviours being shaped by rapid urbanisation, climate change, and globalisation of products and practices, the failure to consider these contexts can limit the potential for high and sustainable impact, despite significant amounts of funding and initiatives targeting health behaviours.

This finding may also be linked to another observation that most studies pertained to fixed-term projects rather than building knowledge around sustainable, long-term projects. Focusing on sustainable projects which can be integrated into existing systems can have a broader and more meaningful impact and enhance opportunities for knowledge and learning. For instance, embedded research models such as learning systems that invest in co-creating and acting on data in the context of ongoing service provision to communities are more fit than one-off short-term projects for improving health long term [36]. This can also create a focus on more sustainable projects that integrate contextual considerations.

On a spatial level, traditional healthcare settings remain the modal context of health behaviour change initiatives followed by community settings. A long-term focus will require modal investments in access to infrastructures, environments and services, that promote behaviour change within the context of communities [37]. There is therefore a need for a more strategic approach to investing in behaviour change initiatives on the continent that aligns initiatives with a coherent view to impact.

We also noticed that there were some disparities in representation among African countries. Almost 64% of African countries were the countries of implementation of identified health behaviour change initiatives. Most of the initiatives were implemented in South Africa (19.8%), Kenya (12.5%) and Nigeria (10.5%). We found no correlation between the number of initiatives and country life expectancy (−0.0029), an insignificant correlation with population size (0.27), a slightly positive correlation with GDP per capita (0.51), and a positive correlation with net GDP (0.68).

This raises the question of why the availability of health behaviour change initiatives is associated with higher gross economic productivity and not a gross impact index such as life expectancy. Previous research also points to substantial disparities within the continent, with Egypt, Nigeria and South Africa accounting for 65.7% of the total research and development spending [37]. This phenomenon can contribute to widening inequities between contexts where there is already more economic capital, and those where there is less.

Public health needs a future-oriented lens with the capacity to look towards what is coming and what is desired, not just what is urgent. As of 2019, the top five leading causes of death in Africa were neonatal conditions (11.3% of deaths), lower respiratory infections (9.9%), diarrhoeal diseases (6.4%), heart disease (5.5%), and HIV/AIDS (5.4%) [16]. It is therefore not strange that 40.7% of behaviour change initiatives across the continent were focused on themes of sexual health and this figure rose to 56.5% in South Africa alone. However, the landscape of health and illness is changing rapidly in Africa. While HIV/AIDS remains the leading cause of death in South Africa, lower respiratory tract infections and tuberculosis which were the second and third leading causes of death have been replaced by ischaemic heart disease and stroke, respectively [38]. As this double burden of NCDs and infections as well as their interlinkages with climate change evolves, investors in health creation must think of how their investment approaches will anticipate future challenges.

Interlinkages between shared disease drivers can be addressed through integrated interventions, which intersect various typologies of behaviour change [39]. Funders can create data systems that collect data across the proximal and distal drivers of behaviours, for example, collecting data not only on hygiene issues, but also on the risks of flooding that contribute to poor hygiene, and on climate vulnerabilities across time and space which increase the prevalence of the floods. Through implementing integrated interventions in close collaboration with grassroots networks which can influence the design, monitoring and sustenance of initiatives [40], investors will be able to make more proactive and sustainable changes. Indeed, past research has found that behaviour change initiatives that incorporated innovative service delivery models, addressed constraints in the environment, and improved inclusive access to services tended to be more effective than others [41].

The research on behaviour change also requires a focus on life-cycle value creation which does not limit knowledge creation to isolated tests of effectiveness but provides a critical understanding of factors around initiative sustainability such as financing, user design [42] and testing, adoption and scaling. Journal editors of peer-reviewed literature and researchers can shift the landscape of research by emphasising networked learning being created in the context of such learning systems or similar institutional and community models. This will encourage more durable and impactful approaches to health behaviour change. Investors in health creation of all kinds, from the public to funders to the private sector must intervene in settings that are more economically deprived to bridge equity gaps. Investors will need to be strategic in their overall portfolio, improving its alignment to actual needs e.g. in terms of life expectancy, quality of life, the burden of disease and other social indicators across the continent. A culture of learning [43] around these elements of initiative design is critical and can happen if the focus of academic documentation is placed on the entire lifecycle of sustainable value creation for health, from start to scale, as against documentation of one-off pilots and trials.

### Limitations

We restricted our analysis to the quantity of health behaviour change initiatives of each type. However, we understand quality is a critical feature of behaviour change initiatives. The scope of our study which reviewed over 300 papers did not allow us to explore this. Future studies may seek to also categorise not only the quantity but the quality of such typologies where data is available. Additionally, given our focus on peer-reviewed literature, our analysis does not include grey literature. Research including grey literature may be instructive. Future research is also needed to explore the relationship between the success of these behavioural initiatives and the GDP of implementing countries. More extensive documentation of long term and integrated initiatives will also be a great contribution to this field of study.

It is important to note also that the lack of representation of some African countries in our analysis does not translate to a lack of health behaviour change initiatives in those countries per se. Some of these policies have also been enacted in Africa. They include the increase in the price of cigarettes in South Africa from 1993 to 2008 which contributed to revenue for social and health sectors [44]. Likewise, several countries including Ghana, Kenya, and the Gambia have laws regulating the advertisement and labelling of food and beverage products, while countries like Botswana have completely banned all television and radio advertising for alcoholic beverages [45]. What appears to be missing is an emphasis on robust evaluation and documentation practices as far as reporting the outcomes of health behaviour change and other initiatives where peer-reviewed knowledge generation priorities are concerned.

In some cases, it was harder to distinguish some downstream initiatives, for example, determining where a downstream initiative was primarily educational, defined as giving information; versus training which focused on building skills. These grey areas may have introduced room for additional errors. To address this potential room for error, we went through multiple rounds of categorisation. First of all, the team was informed about the typology guide and clarified each typology through examples. Unclear cases were discussed by at least two researchers. Multiple papers coded by each team member were reviewed at random to identify any conflict by another team member. This process was continued until no conflicts were identified during a random search. That said, there may have still been some errors in making the distinctions between similar downstream categories. Regardless, since the difficulty lies primarily with education and training initiatives which are both downstream, we can safely conclude that our broader conclusions around the scope of upstream versus downstream initiatives remain valid.

As much as the quantitative data enriched our understanding of interventions targeting health behaviour changes in Africa, the analysis was limited due to the lack of longitudinal evaluations of the interventions analysed. Further, this emphasises that there is also a role for research funders and publishers to play in aligning the research landscape with long-term health creation through integrating immediate responses to urgent health issues with their drivers.

Lastly, with less than two-thirds of African countries being represented in the 300 papers analysed in this review, it might be challenging to generalise the findings of this review to all African countries. This also highlights the lack of interventions in the remaining countries, further emphasising the need to bridge the equity gaps in more economically deprived settings.

## Conclusion

The roots of public health lie in a recognition that behaviour change cannot be sustainably impactful without a strategy that cuts across its individual, social, infrastructural and economic drivers [46]. Investments in behaviour change must reflect this understanding, especially in Africa, a continent with the highest reported burden of HIV/AIDS, tuberculosis and malaria, increasing incidences of preventable non-communicable diseases and the lowest reported life expectancy.

We encourage investors to apply a lens to investing that considers key health trends and outcomes across various countries and channels resources for the desired impact. This means that governments and non-profit funders need to create policies and incentives to encourage partnerships to address health outcomes in underserved contexts [37]. Finance vehicles and enabling policies can support private sector players aiming for impact to invest in demand and supply side factors related to long-term health creation in underserved economic contexts and not just where there is immediate economic opportunity.

For all stakeholders in behaviour change initiatives, it is time to take a strategic, long-term approach to produce, facilitate, and implement more durable and long-lasting health behaviour changes with successful health outcomes. Public health investors in behaviour change, from governments to non-profit funders, to impact investors, will do well to evaluate their portfolios. To what extent does it proportionally reflect multi-component initiatives integrating downstream drivers of behaviours such as education, with their upstream shapers such as infrastructures, enabling services, and policies? To what extent does it consider not just piloting possible interventions, but sustaining them?

Curators of public health knowledge, particularly publishers, will do well to promote a systems approach. To what extent does the literature currently being curated encourage archiving of pilots for which sustenance, scale-up and long-term impact are unclear, over knowledge created through ongoing projects and sustainable learning networks? A strategic approach will shift the kinds of projects emphasised in public health so that the most leverage can be made in the shortest time.

## Biographical note

We are a group of public health practitioners, public health researchers, as well as medical trainees and practitioners with a keen interest, work and lived experience in impactful strategies to effectively improve health and wellbeing in African countries. Our institutions represent actors at the local, national, and regional levels.

## Geolocation information

Nigeria

## Paper context

Our paper investigates the landscape of literature on behaviour change initiatives in Africa to understand the spread of initiatives across typologies, scope, locations, and socioeconomic regions. We found that downstream intervention types, namely education and training, were the most common. We also found a positive correlation between Gross Domestic Product and the number of initiatives identified in each country. This implies the need for durable behaviour change initiatives which also consider intra-regional equity.
